# Comparison of the tumor cell secretome and patient sera for an accurate serum-based diagnosis of pancreatic ductal adenocarcinoma

**DOI:** 10.18632/oncotarget.14449

**Published:** 2017-01-02

**Authors:** Shakhawan Mustafa, Longqiang Pan, Aseel Marzoq, Malak Fawaz, Laureen Sander, Felix Rückert, Andrea Schrenk, Christina Hartl, Rico Uhler, Adem Yildirim, Oliver Strobel, Thilo Hackert, Nathalia Giese, Markus W Büchler, Jörg D Hoheisel, Mohamed Saiel Alhamdani Saeed

**Affiliations:** ^1^ Division of Functional Genome Analysis, Deutsches Krebsforschungszentrum (DKFZ), Heidelberg, Germany; ^2^ Kurdistan Institution for Strategic Studies and Scientific Research, Kurdistan Region, Iraq; ^3^ Chirurgische Klinik, Universitätsmedizin Mannheim, Theodor-Kutzer-Ufer 1-3, Mannheim, Germany; ^4^ Department of General, Visceral and Transplantation Surgery, University of Heidelberg, Heidelberg, Germany

**Keywords:** secretome, pancreatic cancer, biomarkers, cell lines, antibody microarray

## Abstract

Pancreatic cancer is the currently most lethal malignancy. Toward an accurate diagnosis of the disease in body liquids, we studied the protein composition of the secretomes of 16 primary and established cell lines of pancreatic ductal adenocarcinoma (PDAC). Compared to the secretome of non-tumorous cells, 112 proteins exhibited significantly different abundances. Functionally, the proteins were associated with PDAC features, such as decreased apoptosis, better cell survival and immune cell regulation. The result was compared to profiles obtained from 164 serum samples from two independent cohorts – a training and a test set – of patients with PDAC or chronic pancreatitis and healthy donors. Eight of the 112 secretome proteins exhibited similar variations in their abundance in the serum profile specific for PDAC patients, which was composed of altogether 189 proteins. The 8 markers shared by secretome and serum yielded a 95.1% accuracy of distinguishing PDAC from healthy in a Receiver Operating Characteristic curve analysis, while any number of serum-only markers produced substantially less accurate results. Utility of the identified markers was confirmed by classical enzyme linked immunosorbent assays (ELISAs). The study highlights the value of cell secretome analysis as a means of defining reliable serum biomarkers.

## INTRODUCTION

Due to advances in cancer research and medicine, the death rates of several cancer types like lung, colorectal, breast and prostate cancer are decreasing [1]. However, there are also tumor entities for which there is no such improvement. One of them is pancreatic cancer. It is currently the fourth or seventh leading cause of cancer-related deaths in the Western world [2, 3] or China [4], respectively, although only ranked tenth in incidence, and numbers are increasing. Mortality is almost equal to incidence and the average survival period after diagnosis is about five months. This dismal prognosis can be attributed to three major factors. One is the absence of apparent signs and symptoms during early disease stages; consequently, less than 9% of all cases are identified at an early stage of the disease [5]. Second, there is a lack of adequate therapeutic means and the tumors quickly develop resistance to available chemotherapy. Currently, the only effective clinical intervention is surgery, but a mere 10% to 20% of all cases are admissible to tumor resection. Finally, pancreatic cancer exhibits a very early and high rate of metastasis; peritoneal dissemination and liver metastasis are actually the most common cause of death [6].

A major obstacle toward a better prognosis is the absence of reliable and sensitive tools for diagnosis. The available serum biomarkers, such as CEA and CA19-9, are of only limited utility due to a significant lack of specificity and sensitivity [7, 8]. Therefore, the search is on for better performing biomarkers in body fluids for a non-invasive detection of the disease. In a recent report, GPC1^+^ circulating exosomes were described as accurately detecting pancreatic cancer patients [9]. With respect to protein profiles, the screening of PDAC patient sera for suitable biomarkers was reported using recombinant single-chain variable fragment (scFv) binders that target mainly immunoregulatory biomolecules [10]. However, the definition of specific protein biomarkers in blood can be a challenge. One reason is the fact that the origin of the proteins that exhibit variations is not really known [11, 12]. Unless there would be tumor-specific isoforms, proteins could come from all over the body and may not have any direct relation to cancer. Thus, the information could be circumstantial.

Studying the secretome from conditioned media of cultured tumor cells could offer a complementary and well-defined source of molecular information for the discovery of tumor-specific biomarkers (for reviews see [13–16]). The term secretome stands for all proteins that are released from cells into the extracellular space. About 10% of the 22,000 protein-encoding human genes are estimated to encode proteins that are secreted [17, 18]. The secretome is very dynamic in nature and highly sensitive to changes of the overall cellular state, whether at physiological or pathological circumstances. Consequently, analyzing the secretome composition could be instrumental for deciphering the molecular architecture of disease, in particular for a disease as heterogeneous as pancreatic cancer. There have been several reports about an exploration of secretomes for the identification of potential biomarkers [19–23]. A large portion of the secreted proteins – cytokines, hormones or growth factors, for example – are present at very low levels [18]. Therefore, sensitivity and resolution of the analysis processes are limiting. In serum analyses, the problem of low abundance is actually magnified by the presence of large quantities of albumin and globins, which can obscure an analysis of rare proteins or mask their presence altogether. Depletion of highly abundant proteins is not a solution, since their removal does affect the abundance and relative ratios of the other proteins, too [12]. To circumvent these problems, immunofractionation with appropriate antibodies is often applied prior to analysis. To gather enough protein in such a process, rather large sample volumes are required. Also, quantification is difficult to achieve since different antibody affinities lead to different yields during the purification process and normalization processes are not easily applicable. In addition, a translation into clinical practice is difficult to achieve.

Using immunoassays directly for detection rather than just as a means of purification offers an alternative that could circumvent many of the above limitations [13]. Antibody microarrays have already been used for the measurement of the abundance of cytokines and growth factors in both conditioned media of cultured cells and human body fluids [24–27]. Many of these studies made use of a sandwich format: one antibody is attached to the array surface and isolates the target protein from the samples; a second antibody that binds to a different epitope of the same protein is subsequently applied for labeling purposes. Antibody cross-reactivity prevents the parallel measurement of more than a few tens of proteins [28, 29], however. For a large-scale screening for protein markers, direct labeling of protein extracts prior to incubation is therefore advantageous, allowing the simultaneous analysis of thousands of proteins with sensitivities of attomolar concentrations by routine processes [30, 31]. Serum albumin and globins do not affect the analysis [32]. The result of the screening process could be transferred quickly to a clinical setting in a simplified format, once a signature has been defined. For clinical use, a sandwich format, for example an enzyme linked immunosorbent assay (ELISA), could be applied, since the number of target molecules is limited to a few.

Utilizing an antibody microarray, we analyzed the secretomes of a panel of pancreatic tumor and non-tumorous cell lines in order to identify proteins that could act as biomarkers of disease. Taking advantage of the results, we studied serum samples of patients with pancreatic ductal adenocarcinoma (PDAC) and chronic pancreatitis (CP) as well as sera from healthy donors for the establishment of a set of defined biomarkers. The serum analysis was performed on independent training and test sets of patient sera, which were studied on two distinct formats of antibody microarrays, respectively. The potential of translating the identified marker signature into a clinical format was demonstrated by subsequent validation with commercial ELISA kits.

## RESULTS

### Basic quality assessment of cell secretome analysis

The secretome samples of human dermal fibroblasts, six primary and ten established pancreatic tumor cell lines were studied. The last group was selected from a panel of 24 pancreatic cancer cell lines, whose intracellular proteomes had been studied with the same antibody microarray setup [33]. The established cell lines represent tumors that had originally been isolated from both male and female patients and also differ in their anatomic origin (primary tumor, metastasis and ascites) and degree of differentiation. The primary tumor cells were also from patients of both genders and represent metastases or primary tumors; the latter had been located in either the head or tail of the pancreas. This and other information about the tumor cells that were used in the analysis is provided in Table 1.

Table 1Cell lines used in the study

**Table:** 

Description of established cell lines														
Cell line	Patient gender			Cell source		Histology						Grade	Age (years)	
A818-1	Female			Ascites		Moderately differentiated, ductal adenocarcinoma						G2	75	
AsPC-1	Female			Ascites		Well-moderately differentiated, ductal adenocarcinoma						G2	62	
BxPC-3	Female			Primary tumour		Moderately differentiated, ductal adenocarcinoma						G2	61	
CFPAC-1	Male			Liver metastasis		Well differentiated, ductal adenocarcinoma; cystic fibrosis						-	26	
Colo357	-			Lymph node metastasis		Well differentiated, ductal adenocarcinoma						G2	-	
MIA PaCa-2	Male			Primary tumour		Poor-moderately differentiated, ductal carcinoma						G3	65	
PaCa-44	Female			Primary tumour		Moderately differentiated, ductal adenocarcinoma						G2	44	
PANC-1	Male			Primary tumour		Poorly differentiated, ductal epithelioid carcinoma						G3	56	
Pt45P1	-			Primary tumour		Moderately differentiated, ductal adenocarcinoma						G3	-	
SK-PC-1	Male			Primary tumour		Well differentiated, ductal carcinoma						-	-

**Table:** 

Clinical features of the primary pancreatic cancer cell lines														
Cell line		Patient gender	Tumour localization		Histology		Classification				Postoperative survival (days)			Patient status
							**T**	**N**	**M**	**G**				
PacaDD119		Male	Pancreas head		Poorly differentiated, ductal adenocarcinoma		3	1	0	3	445			Dead
PacaDD137		Female	Pancreas head		Moderately differentiated, ductal adenocarcinoma		2	0	0	2	478			Alive
PacaDD159		Male	Pancreas tail		Moderately differentiated, ductal adenocarcinoma		3	0	0	2	169			Dead
PacaDD135		Female	Liver metastasis		Moderate-poorly differentiated, ductal adenocarcinoma (partiallly mucinous)		-	-	Hep	2	66			Dead
PacaDD161		Female	Liver metastasis		Poorly differentiated, adenocarcinoma		-	-	Hep	3	-			Dead
PacaDD183		Female	Pancreas head		Moderate differentiated, ductal adenocarcinoma		-	-	-	2	55			Dead

The secretomes of the different cells were labeled with fluorescent dye and mixed individually with a common reference sample. This reference sample was made by pooling the intracellular proteomes of all the cell lines, from which the secretomes were isolated, and labeled with another dye. Analysis was performed with an antibody microarray targeting 735 proteins (for a complete list see Supplementary Table 1) [33]. The samples produced a very similar data quality even without normalization. Taking into consideration all antibody spots on the microarray – 3010 due to multiple spotting – without applying any filtering, the number of spots on which there was a signal-to-noise ratio (SNR) larger than two times the standard deviation of the average background signal at this location was higher than 93% for the common reference and varied around 80% for the incubations of the secretome samples (Supplementary Figure 1A). For individual proteins, signal-to-noise ratios as high as 100 were observed; the mean across all proteins with signals above background was 4.38 (±7.68). Data normalization yielded highly comparable results (Supplementary Figure 1B). The intra- and inter-array coefficients of variation across a large number of microarray production batches ranged between 13% and 20%.

### Variations of protein abundance in cancer cell secretomes

The comparison of the secretomes of the 16 pancreatic cancer cell lines and non-cancerous fibroblast cells yielded 112 differentially abundant proteins (Supplementary Table 2). All 112 proteins were similarly up- or down-regulated in at least 14 of the investigated cancer cell lines. Analyzing the annotations of the proteins revealed that the majority of them are prominently associated with the functional categories of “cellular growth and differentiation”, “decreased apoptosis and cell death” as well as “increase in organismal death and cancer” (Table 2 and Supplementary Table 3). This result indicates a potential role of secreted proteins as anti-apoptotic factors that could be involved in the control of the maintenance and survival of pancreatic tumor cells and reflects the bad prognosis associated with the tumor. In addition, the secretome profiles predict an influence of PDAC tumor cells on regulating immune cells and their quantity within the tumor, suggesting that tumor cells could control the trafficking of immune cells, such as phagocytes, monocytes and neutrophils, into the tumor microenvironment.

**Table 2:** List of the most frequently predicted functions associated with the secretome of pancreatic cancer cell lines

Function annotation	Predicted activation state	Prediction (z-score)	p-Value	Proteins	Number of proteins
Cell death of pancreatic cancer cell lines	Decreased	-2.568	4.59 E-07	ALB, CXCL8, DDIT3, FN1, MTOR, RPS19, SPP1, TGFB1	8
Apoptosis of pancreatic cancer cell lines	Decreased	-2.365	2.27 E-06	ALB, CXCL8, DDIT3, FN1, MTOR, RPS19, TGFB1	7
Quantity of phagocytes	Increased	+2.049	3.53 E-12	BCL2A1, CNN2, CXCL8, DCN, FABP1, FASTK, FPR1, GJA1, ID1, IL10, IL1A, IL2, S100A8, SELE, SELL, SPP1, TGFB1, TGFBR2, TIA1, VCAM1	20
Quantity of neutrophils	Increased	+2.156	1.23 E-10	CNN2, DCN, FASTK, FPR1, GJA1, ID1, IL10, IL1A, S100A8, SELE, SELL, TGFB1, TIA1, VCAM1	14
Organismal death	Increased	+2.174	1.49 E-15	AKR1C3, APC, ATP6AP1, BCL2A1, BGN, CTTN, CXCL8, DAB2, DCN, DDX17, EIF2AK4, EPHB3, FN1, FOS, GAS1, GJA1, GPM6B, HNRNPC, HSP90B1, ID1, IL10, IL2, IMPDH2, KLF4, LAMTOR1, MAPK3, MLH1, MME, MMP2, MTOR, NCOR2, NUSAP1, PAX2, POU2F1, PVRL1, RPS19, RPSA, S100A8, SELE, SERPINB5, SLC19A1, SPINT2, SPP1, TCEA1, TGFB1, TGFBR2, TIA1, TIE1, TJP2, TOP2A, TPT1, UBC, VCAM1, ZBTB17	54
Quantity of monocytes	Increased	+2.256	1.85 E-08	CNN2, CXCL8, GJA1, ID1, IL10, IL1A, SELE, SELL, VCAM1	9

The prediction z-score (plus for activation and minus for inhibition) was calculated within IPA software as described in the Materials and Methods section. The complete list can be found as supplemental information (Supplementary Table 3).

Interestingly, there was little difference between the secretomes of the primary and established pancreatic cancer cell lines. In comparison to other tumor-relevant cells, such as stellate cells or macrophages, the secretomes were rather different, however (unpublished data). Also the location, from which the cells had originally come from (primary tumor; ascites; metastases in the liver; metastases in the lymph node), did not make a difference, and neither did tumor grade or the degree of differentiation. This is in contrast to the results of an analysis of the cells’ intracellular proteomes, in which significant variations have been observed [33]. Four of the six primary tumor cell lines were basically identical, while the other two – isolated from mucinous PDAC and a long-term survivor, respectively – showed slightly more but nevertheless little variation. Some of the proteins that were regulated in the tumor cell secretome had been reported to be present in body fluids, such as saliva, cerebrospinal fluid, tears, blood or urine.

### Comparison of secretomes to the related intracellular proteomes

**Figure 1:**
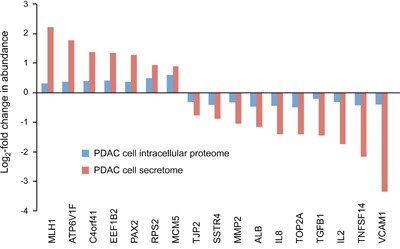
Proteins that are commonly regulated in secretome and intracellular proteome of tumor cells The proteins are listed, which exhibited similar regulation in both secretome and proteome analyses of the ten established pancreatic cancer cell lines in comparison to non-tumor cells. The bar sizes indicate the relative degree of regulation.

We have previously studied with the very same antibody microarray the intracellular proteomes of the PDAC cell lines whose secretome data are reported here [33]. Comparing the profiles, the majority of changes in abundance were unique to either the intracellular proteome or the secretome. Only 17 proteins were similarly regulated between the two sample types (Figure 1). This represents 15% (17/112) of the secretome-specific and 13% (17/132) of the intracellular proteome variations, or some 2% (17/735) of all studied proteins. Most of the 17 proteins are actually associated with extracellular functions, according to gene ontology (GO) terms, or are sheded components of the plasma membrane. All exhibited a substantially larger abundance variation in the secretome than intracellularly. For the large majority of proteins, however, the expression patterns were different in secretome and intracellular proteome. For example, most cytokines were found up-regulated in the secretome and down-regulated intracellularly, while the opposite was observed for nuclear proteins like RPS19, NCL and BRPF3.

### Protein variations in patient serum samples

To investigate the impact of secretome data on the quality of a patient diagnosis based on serum protein data, serum samples from patients were studied. Two independent sample groups, a training and test set, were used in this analysis. The training set consisted of sera from 47 PDAC patients, 18 people with chronic pancreatitis (CP) and 27 age- and sex-matched healthy individuals. The test set was composed of 25, 25 and 22 serum samples, respectively. The characteristics of the patients and donors are summarized in Table 3. Two distinct antibody microarrays were used for analysis. The training samples were studied with the antibody microarray of 735 antibodies already used in the secretome analysis. The test group was incubated on a larger array containing about twice the number of antibodies (Supplementary Table 4). However, several antibodies of the smaller microarray were missing on the larger array as supplies had run out and could not be replaced.

**Table 3:** Characteristics of healthy donors and patients from whom serum samples were collected

Healthy donors	
Age: range (average)	Sample set 1: 39-71 (52)Sample set 2: 35-74 (55)
Gender: male/female	Sample set 1: 0.60Sample set 2: 0.62
**CP patients**	
Age: range (average)	Sample set 1: 25-76 (44)Sample set 2: 24-78 (51)
Gender: male/female	Sample set 1: 0.70Sample set 2: 0.85
**PDAC patients**	
Age: range (average)	Sample set 1: 39-74 (58)Sample set 2: 38-81 (61)
Gender: male/female	Sample set 1: 0.66Sample set 2: 0.67
**Tumour localisation**	**% of cases**
Pancreas head	55
Pancreas body	14
Pancreas tail	3
Papilla vateri	10
Multiple	18
**Grading**	**% of cases**
G2	48
G3	34
unknown	18
**R classification**	**% of cases**
R0	55
R1	38
R2	3

**Figure 2:**
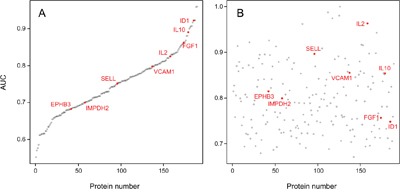
AUC values of the 189 individual serum markers Analysis by Receiver Operating Characteristic (ROC) curves was performed for all identified serum protein markers individually. Panel A shows the result calculated from the training set; the respective AUC values are shown, ranging from 55.2% to 96.0%. In panel B, the AUC values are shown as calculated for the individual marker molecules in the test set. For presentation, the order of the markers along the x-axis was kept as in panel A, highlighting the limited degree of reproducibility for individual markers.

Looking at the individual diagnostic accuracy of the 189 proteins that were found to be differentially abundant in serum of PDAC patients and healthy donors (Supplementary Tables 5 and 6), they differed in their ability to discriminate between the two groups as indicated by their individual AUC values. In the test set, rather good accuracy values could be determined for some of the proteins (Figure 2A). When controlling these performances in the training set, however, none exhibited an overall accuracy that would be sufficient individually (Figure 2B).

**Figure 3:**
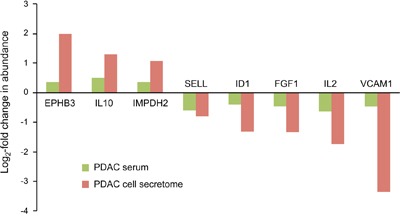
Proteins that were similarly regulated in both tumor cell secretome and PDAC patient sera Of the 112 differentially expressed proteins in the tumor cell secretome compared to the non-tumorous cells, only 8 were similarly regulated in the serum. The red and green bars indicate the degree of regulation in secretome and PDAC serum, respectively.

Of the 112 proteins found to vary in the secretome of tumor cells, only 8 proteins were similarly regulated in serum samples of PDAC patients, but not exhibiting different abundance in CP sera (Figure 3). In all cases, the variation was substantially bigger in the secretome than in the serum. Since it is likely that a tumor's secretome gets diluted and in part obscured by the proteins that are secreted by cells in other organs and tissues, such a difference is expected. The expression of most proteins was either unchanged in one or both sample types or varied even inversely in secretome and serum.

### Serum-based diagnostics

**Figure 4:**
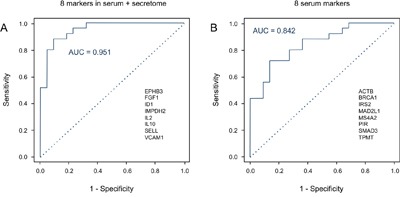
Diagnostic potential of the protein signatures in serum **A**. A Receiver Operating Characteristic (ROC) curves was calculated for the signature of 8 proteins that exhibited shared expression changes in the analyses of PDAC serum and secretome, yielding an AUC value of 95.1%. The protein names are shown. **B**. In comparison, the ROC curve is presented for the best performing panel of 8 proteins that differed in abundance in the serum only; AUC is 84.2%. The relevant proteins are listed.

The 8 proteins that similarly varied in abundance in PDAC sera and in tumor cell secretomes – EPHB3, FGF1, ID1, IL2, IL10, IMPDH2, SELL, VCAM1 – did not exhibit a superior performance as markers individually (Figure 2). When applied as a signature for PDAC diagnosis, however, a Receiver Operating Characteristic (ROC) curve analysis yielded an accuracy – expressed as area under the curve (AUC) value – of 95.1% for distinguishing PDAC from healthy (Figure 4A). For comparison, the best performing panel of 8 marker molecules, which were differentially abundant in sera but not in the secretome, was selected based on the training set. Applied to the test set, they produced an AUC value of 84.2% (Figure 4B). Even applying more than 8 of the proteins that showed variation in serum abundance only, no better distinction of sera from cancer and healthy patients could be achieved. This documents that the information content of the signature based on molecules that varied in both secretome and serum is significantly higher than that derived from serum-only markers.

The 8 markers shared between PDAC cell secretome and PDAC patient sera were not able to separate PDAC from CP sera, however. In this context, they performed basically identical to the best serum-only marker signature with AUC values of 71.2% and 72.2%, respectively. For discriminating PDAC from CP sera, the most informative signature was made up of 25 proteins, yielding in a ROC analysis an AUC value of 97.1% (Supplementary Figure 2).

### Validation by ELISA

**Figure 5:**
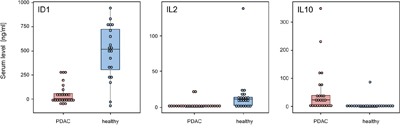
ELISA validation of identified marker proteins The protein content was analyzed of 25 and 21 sera, respectively, isolated from PDAC patients and healthy blood donors. Proteins ID1 (A), IL2 (B), and IL10 (C) were studied with commercially available assays. All markers showed a highly significant degree of variation of abundance, with p-values < 0.001.

Complex antibody microarrays like the ones used here are unlikely to be utilized in clinical routine. An analysis would produce information that is not required for diagnosis. Also in terms of robustness, other methods are superior and already established in a clinical setting. Therefore, we confirmed our results not just by applying a scheme of independent training and test samples but used commercial ELISA kits for ID1, IL2 and IL10 in addition. The antibodies of the three ELISA kits were different from the molecules on the microarrays used to define the diagnostic signature. ID1, IL2 and IL10 were selected at random from the eight proteins that define the signature. An analysis of all eight was not possible, since the amount of serum was limiting. While a protein amount equivalent to about two microliters of serum is sufficient for a microarray analysis that studies as many antigens as there are antibodies on the microarray, the protein content of 50 μl to 150 μl serum was required for each individual ELISA. In total, we analyzed 25 and 21 sera from PDAC and healthy control samples, respectively. In agreement with the microarray data, both ID1 and IL2 were found significantly lower in PDAC sera as compared to healthy control samples and IL10 was present at a significantly higher level (all three with p < 0.001) (Figure 5).

## DISCUSSION

Blood-based diagnosis could improve the outcome of therapy, even for a disease as deadly as PDAC. Applied to people, who are at risk of developing pancreatic cancer – for instance, patients who have undergone tumor resection, familial cases, individuals with particular genetic syndromes or predisposing diseases, such as chronic pancreatitis or diabetes – an improvement in diagnostic accuracy could have significant consequences on life expectancy and quality. Analyses of peripheral blood are predestined to such an end; studies with microRNA, circulating DNA and exosomes or proteins have been reported [9, 10, 34–37]. We set out to study the secretome of tumor cells in order to improve accuracy of protein-based diagnosis. A protein profile should represent the pathological condition specifically as it reflects the actual changes in the activity of diseased cells. In the analysis, a large panel of antibodies was applied. Many target proteins that are associated with cancer according to KEGG and GO term annotation and had demonstrated their diagnostic power for other tumor forms [32, 38] and in an analysis of the intracellular proteome of 24 pancreatic tumor cell lines [33]. An earlier, mass spectrometry based analysis of conditioned media of six tumor cells had reported 63 most differentially abundant molecules [23]. No overlap was found between them and the 112 molecules that we identified. This is not too surprising given the many differences between the studies. The criteria for selecting differentially abundant proteins were different; sample preparation was also very different. In addition, only about 15% of all detected proteins overlapped between the two studies. The reasons for this are the facts that the microarray analysis was biased by the availability of antibodies, but that its detection sensitivity is substantially higher. Therefore, the results are likely to be complementary.

The fact that there was very little correlation of protein abundance in secretomes and the related intracellular proteomes suggests that the regulation of protein secretion occurs independent of the intracellular protein expression. Similar data, focusing on the protein content of exosomes released from colorectal cancer cells showed also a nearly inverse protein abundance in exosomes and intracellularly [39]. In view of these results, a transfer of tumor markers that were identified in tissue cells for an identification of disease in serum does not seem promising. The functional annotations of the 112 proteins that were found to differ in the secretomes of tumor and non-tumorous cells indicated a potential role of secreted proteins as anti-apoptotic factors. Also, the results suggested an influence on immune cells. It is likely that several properties of the tumor are mediated by secretory factors released by the various cell types in the tumor microenvironment, of which tumor cells are only a part. We are currently pursuing this line of research further toward a better understanding of the regulative networks created between microenviromental cells by means of secreted proteins.

Surprisingly, the secretome analysis did not reveal any apparent correlation of secretome composition with the tumor cells’ original location – primary tumor, metastatic tumor or ascites – or the degree of differentiation, while the intracellular proteomes had shown significant differences [33]. This suggests that it is unlikely to detect informative variations of this type in serum either, assuming that the serum acts as a combined representation of the various cell secretomes. However, other cells of the respective microenvironment of primary tumor, metastatic tumor or ascites may well secrete protein signatures, which differ in their composition, and may permit serum-based diagnosis.

The 8 proteins that exhibited similar abundance differences in serum and secretome profiles belong to the proteins that are most strongly regulated in terms of their abundance level in tumor secretomes. One could speculate that they are therefore the ones most likely to remain detectable after becoming part of the serum. Other proteins may loose their discriminative power as they get diluted and obscured too much so as to remain informative. While for understanding tumor biology the identification of even subtle variations could be as important as that of large changes in expression, it could be advantageous for serum diagnostics to concentrate on proteins, which in secretome analyses are present at high concentrations and exhibit large differences in expression.

The value of comparing secretome analyses and serum studies for the definition of reliable biomarkers is further demonstrated by the results obtained for protein GRPR, for example, which was implicated as a marker molecule [40]. In view of contradictory results, with GRPR being up-regulated in tumor cell secretome while down-regulated in PDAC patient serum, the reliability of the marker could be questioned. It cannot be excluded that it is a bona fide marker of high predictive value nevertheless and that the serum abundance reflects the overall release of the protein from other cell types in or around a tumor. However, as long as this is not evaluated in more detail, one should act carefully with utilizing GRPR as a marker in liquid biopsy.

Diagnosis on basis of the sera yielded a signature of just eight proteins, which permitted the identification of PDAC patients with an accuracy of 95.1%. Some of the proteins, such as IL10, ID1 and IL2, had been implicated as possible biomarkers of pancreatic cancer before [41–43]. However, the result sheds new light on the process by which marker molecules should be selected for a useful diagnostic signature. Compared to the other 181 serum markers, the eight molecules shared between secretome and serum do not show any particular discriminative performance individually. In combination, however, they beat any panel of eight markers made only from serum proteins by quite a margin. Actually, even adding up more serum markers could not yield a signature with a better diagnostic performance. In the comparison of PDAC and CP sera, the eight proteins were again not different from other markers individually. But also as a signature, they were not superior to other panels. The reason for this could be the fact that they were identified in a secretome analysis, in which tumor and normal cells were compared to each other. This suggests that adding to a signature the apparently best individual markers may not yield the best possible signature. Instead, considering biological and functional data, such as variation in the secretion from different cell types, could contribute valuable information for selecting useful markers.

The results of this study do not represent a diagnostic tool as yet. To such ends, more is needed, such as a broader analysis of robustness and an assessment of specificity in other diseases. However, the data shown here are an initial step of identifying suitable biomarkers of high accuracy and highlight the fact that an analysis of cell secretomes can be enormously helpful in this identification process.

## MATERIALS AND METHODS

### Reagents and antibodies

All chemicals used in this study were purchased from Sigma-Aldrich (Munich, Germany), unless stated otherwise, and were of highest purity or protein grade. Two distinct antibody microarrays were used. The first one was composed of 735 antibodies. The experiments with the cell secretomes and the first set of serum samples were performed on this microarray. It had been used also for earlier studies of sera [32] and PDAC cell proteomes [33]. For validation with the second set of serum samples, a newly designed microarray was utilized that consisted of 1439 antibodies. A large bulk of the antibodies was produced by Eurogentec (Seraing, Belgium). The others were purchased from various sources or provided by collaborating partners. A complete list of the arrayed antibodies and their target proteins is given in Supplementary Tables 1 and 4, respectively.

### Cell culture and secretome collection

All cells used in this study were checked for contamination with mycoplasma before and after cell growth. The pancreatic tumor cell lines A818-1, AsPC-1, BxPC-3, CFPAC-1, Colo357, MIA PaCa-2, Paca44, PANC-1, Pt45P1 and SK-PC-1 and six primary PDAC cell lines were used in the analysis (Table 1); the primary cells have been described in detail previously [44]. Normal human dermal fibroblasts (NHDFs) (PromoCell, Heidelberg, Germany) acted as control. All cell lines but the fibroblasts were cultured in Iscove's Modified Dulbecco's Medium (IMDM) (Invitrogen, Darmstadt, Germany) containing 10% fetal bovine serum, 100 U/ml penicillin and 100 μg/ml streptomycin at 37°C in a humidified atmosphere of 5% CO_2_ and 95% air. The fibroblasts were grown in PromoCell fibroblast growth medium. Cells were cultured to 85-90% confluency; then the medium was removed and the cells were washed three times with phosphate-buffered saline (PBS) followed by two washes with serum-free growth medium. Subsequently, the cells were incubated in serum-free growth medium for 12 h in order to synchronize cell growth. The medium was replaced and cells were incubated for another 48 h. Then, the medium was collected, centrifuged at 3500 g for 10 min, filtered through 0.22 μM nylon filters and stored at -80°C.

### Collection and handling of serum samples

For all samples analyzed, written informed consent was given by the patients and healthy donors. Ethical approval was obtained from the local ethics committee at the University of Heidelberg; ethics vote 159/2002 of 28 December 2007. Two groups were selected and analyzed separately as training and test sets. For the training set, serum samples were collected via venipuncture from 47 patients with pancreatic ductal adenocarcinoma (PDAC), 18 people with chronic pancreatitis (CP) and 27 age- and sex-matched healthy individuals. The test set was composed of 25, 25 and 22 serum samples, respectively. Patient diagnosis was based on histological analyses. The disease stage was determined by classification of tumor, node and metastasis (TNM). Exclusion criteria had been applied to patients, who had secondary conditions such as autoimmune, inflammatory or infectious diseases. The clinical characteristics of all subjects are shown in Table 3. The serum samples were stored immediately at -80°C until use.

### Preparation of the conditioned media

Three different methods were initially used for concentrating the protein content of cell media: trichloroacetic acid (TCA) precipitation, dialysis with lyophilization and ultrafiltration. In our hands, ultrafiltration was found to be the best method for obtaining protein in high quality with respect to reproducibility, concentration and integrity. Vivaspin-20 tubes with a molecular weight cut-off value of 3 kDa (Sartorius, Göttingen, Germany) were used according to the manufacturer's protocol. Each tube was filled with 20 ml medium and spun at 5,000 g at 4°C for 2 h in a swing-bucket Varifuge 3.0R Heraeus centrifuge (Thermo Fisher Scientific, Bonn, Germany). Twice, a desalting step was performed using 20 ml of 0.1 M bicine (pH 8.5) at the same centrifugation conditions. The protein concentration of the ultrafiltrated solution was determined with the bicinchoninic acid (BCA) protein assay reagent kit (Thermo Fisher Scientific). Protein integrity was checked by SDS gel electrophoresis. Samples were kept frozen at -80°C until use.

### Samples preparation and protein labeling

The protein concentration of the serum samples or the secretome protein isolates was adjusted with 50 mM bicine buffer (pH 8.5) to 1.0 mg/ml. Proteins were labeled with the fluorescent dyes DY649 or DY549 (Dyomics, Jena, Germany), respectively, at a molar dye/protein ratio of 7.5 in 50 mM bicine buffer (pH 8.5) in the dark at 4°C for 2 h. Unreacted dye was quenched by the addition of 10% glycine in 50 mM bicine buffer (pH 8.5).

As a common reference sample, we utilized the pooled cellular protein lysates of all tested cell lines as described previously [33]. In brief, after collecting the growth medium for secretome analysis, the respective cells were washed with PBS and layered with lysis buffer composed of 50 mM bicine buffer (pH 8.5) containing 20% glycerol, 1.0 mM MgCl_2_, 5.0 mM EDTA, 1.0 mM phenylmethanesulfonyl fluoride, 1.0 U/ml benzonase (Merck Biosciences, Schwalbach, Germany), Halt protease and phosphatase inhibitor mixture (Thermo Fisher Scientific), 0.5% Nonidet P-40 substitute, 1.0% cholic acid, 0.25% *n*-dodecyl-β-maltoside (Genaxxon Bioscience, Ulm, Germany) and 0.5% amidosulfobetaine-14, and kept at 4°C for 30 min. The cells were collected with a cell scraper and passed through a fine needle multiple times to completely disrupt them. Samples were centrifuged at 20,000 g and 4°C for 30 min in order to pellet the debris. The supernatants were collected and pooled in identical amounts. This protein reference sample was labeled with the dye DY549 for analyses of secretome samples labeled with DY649, and vice versa. For serum analyses, a pool reference was prepared in an identical manner from a pool of all serum samples.

### Antibody microarrays production

A protocol was used, which has been described in very detail before [33, 45]. In brief, the antibodies were spotted on epoxysilane-coated slides (Nexterion-E; Schott, Jena, Germany) using the contact printer MicroGrid-2 (BioRobotics, Cambridge, UK) and SMP3B pins (Telechem, Sunnyvale, USA) at a humidity of 55-65%. The printing buffer was composed of 100 mM bicine buffer (pH 8.5) containing 0.005% Tween-20, 0.05% sodium azide, 5% trehalose, 5 mM magnesium chloride, 137 mM sodium chloride, and 1 mg/ml of the respective antibody. Each antibody was spotted in quadruplicates. Positional marker molecules as well as negative and housekeeping controls were included on all microarrays. After the actual printing process, the slides were allowed to equilibrate at room temperature and 55-65% humidity overnight. They were then stored in dry and dark conditions at 4°C.

### Antibody microarray analysis

Incubation of arrays was performed as previously reported [45]. Prior to incubation with labeled samples, the printed arrays were equilibrated at room temperature for 30 min followed by washing twice with PBS containing 0.05% Tween-20 (PBST). Arrays were blocked with 5 ml of 10% non-fat dry milk (Bio-Rad, Munich, Germany) in PBST at room temperature for 3 h using Quadriperm chambers (Greiner Bio-One, Frickenhausen, Germany). The blocked slides were incubated with 35 μg each of a labeled secretome sample and the pool reference in 5 ml of 1% milk in PBST in the dark, constantly shaking at 4°C overnight. For serum analyses, 25 μg protein were used. After the incubation, the slides were washed five times for 5 min with PBST and 10 min with distilled water, constantly shaking at 150 rpm. The microarrays were dried in a ventilated oven at 37°C. A Tecan PowerScanner system (Tecan, Männedorf, Switzerland) was used for image capture. Scanning was done at constant laser power and photo-multiplier tube gain. The resulting images were analyzed with the software package GenePix Pro 6.0 (Molecular Devices, Sunnyvale, USA).

### Enzyme linked immunosorbent assay (ELISA) analysis

ELISA testing was performed for proteins ID1, IL2 and IL10 by means of the respective ELISA kit of Abcam (Cambridge, UK) according to the manufacturer's instructions. Serum samples from the test set were used for analysis.

### Data analysis

Statistical analysis of microarray data was conducted with the Chipster software package (v1.4.6, CSC, Finland). The data were presented as the median of the signal intensities in the red (DY-649) and green (DY-549) channels, respectively. The coefficient of variance for the pool reference was less than 10% across all tested microarrays. Signal-to-noise ratio (SNR) was calculated as (median foreground vs. median background) / (standard deviation of background) for both the red and green channel. The Loess approach was used for data normalization with background correction offset (0, 50) of the normexp [46]. Two-group test between normal and cancer cells was done using the empirical Bayes test with Bonferroni-Hochberg adjustment for multiple testing [47]. A p-value of 0.05 or less was considered significant. Array quality was assessed using the ordinate method Detrended Correspondence Analysis [48]. In addition, array results were clustered using their Pearson correlations and a dendrogram was constructed using the Average Linkage method.

For a prediction of the functional aspects of the differentially expressed proteins, the Ingenuity Pathways Analysis (IPA) software package (version 6.3; Ingenuity Systems, Redwood City, USA) was applied. Prediction of function activation of inhibition was calculated within IPA using z-score method. Component annotation was mapped using the web-based Gene Ontology tool of UniProt (www.uniprot.org). The Ingenuity software also permitted a literature analysis with respect to the biomarker status of particular proteins.

The sensitivity and specificity of discriminating patient groups were calculated with support vector machine (SVM) algorithms in R programming [49] with a threshold level of zero. The samples were divided into a training set and test set. Using the SVM decision values, a receiver operating characteristics (ROC) curve and the respective area under the curve (AUC) value were calculated. To define biomarker signatures, a leave-one-out cross-validation procedure was applied. A linear kernel was used and all the other parameters were set as default to avoid overfitting. Each time one sample is removed from the training set, the remained samples were analyzed as follows: each protein in the remained samples was removed in turn, the remaining protein groups were analyzed with Wilcoxon test. The most significant group was chosen and used for calculating a SVM decision value with the left-out sample. The same strategy was used with the chosen group until only one protein is left. By this approach, a candidate biomarker list was found, which was then evaluated with the test set.

## SUPPLEMENTARY MATERIALS FIGURES AND TABLES














