# Supplementary material for: Comparison of the tumor cell secretome and patient sera for an accurate serum-based diagnosis of pancreatic ductal adenocarcinoma
Source: Oncotarget. 2017 Jan 2;8(7):11963–76. doi: 10.18632/oncotarget.14449 (PMC5355318; doi:10.18632/oncotarget.14449)
Supplement: Supplementary file 1 [file oncotarget-08-11963-s001.pdf]

Comparison of the tumor cell secretome and patient sera for an accurate serum-based diagnosis of pancreatic ductal adenocarcinoma

SUPPLEMENTARY FIGURES AND TABLES

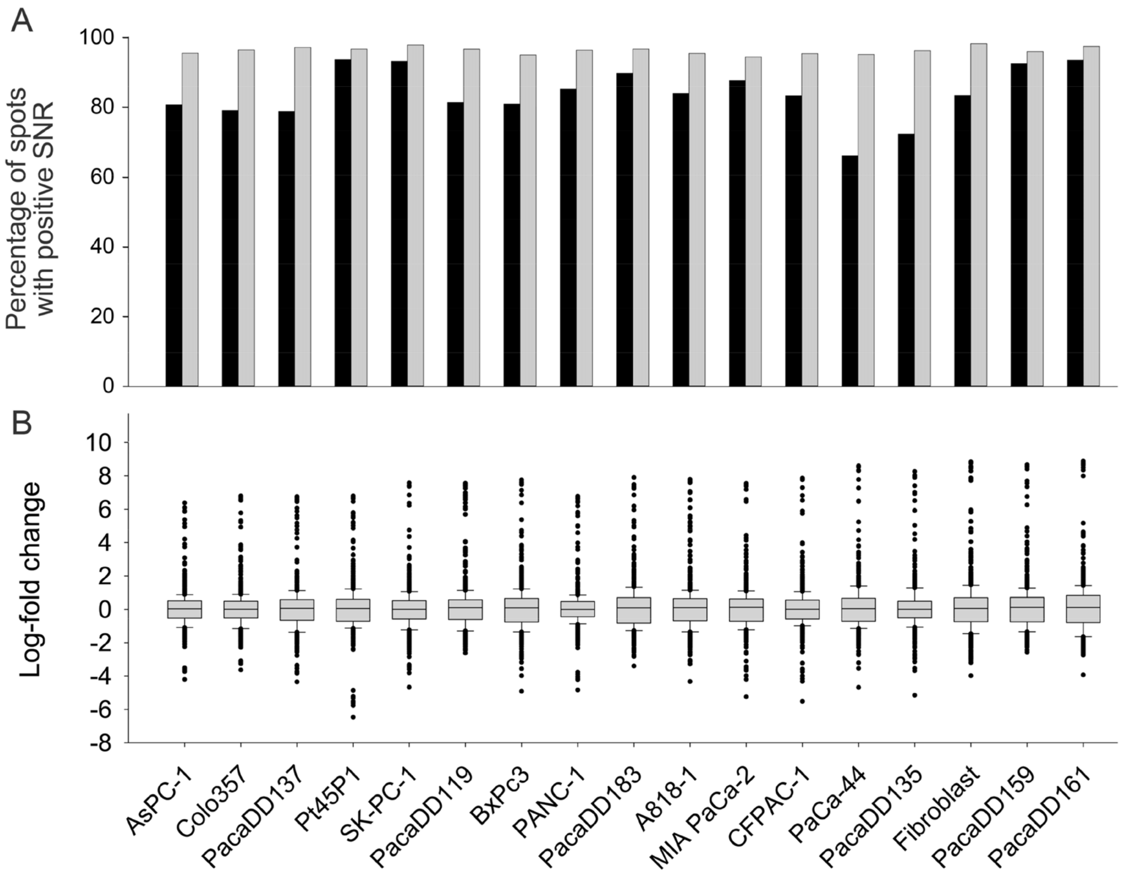

Supplementary Figure 1: Quality assessment of microarray analyses. A. The median percentage of microarray antibodies is shown that exhibited a signal that was significantly above background. The black bars represent the results with the intracellular proteins used as common reference; the grey bars stand for the secretome samples. The order of the cells was defined by the amount of protein that was obtained from the secretome, from relatively high (left) to low amounts (right). As can be seen, there was no apparent influence of the protein preparation yield. **B.** A box plot representation is shown of all signal intensities of the secretome samples normalized to the pool reference.

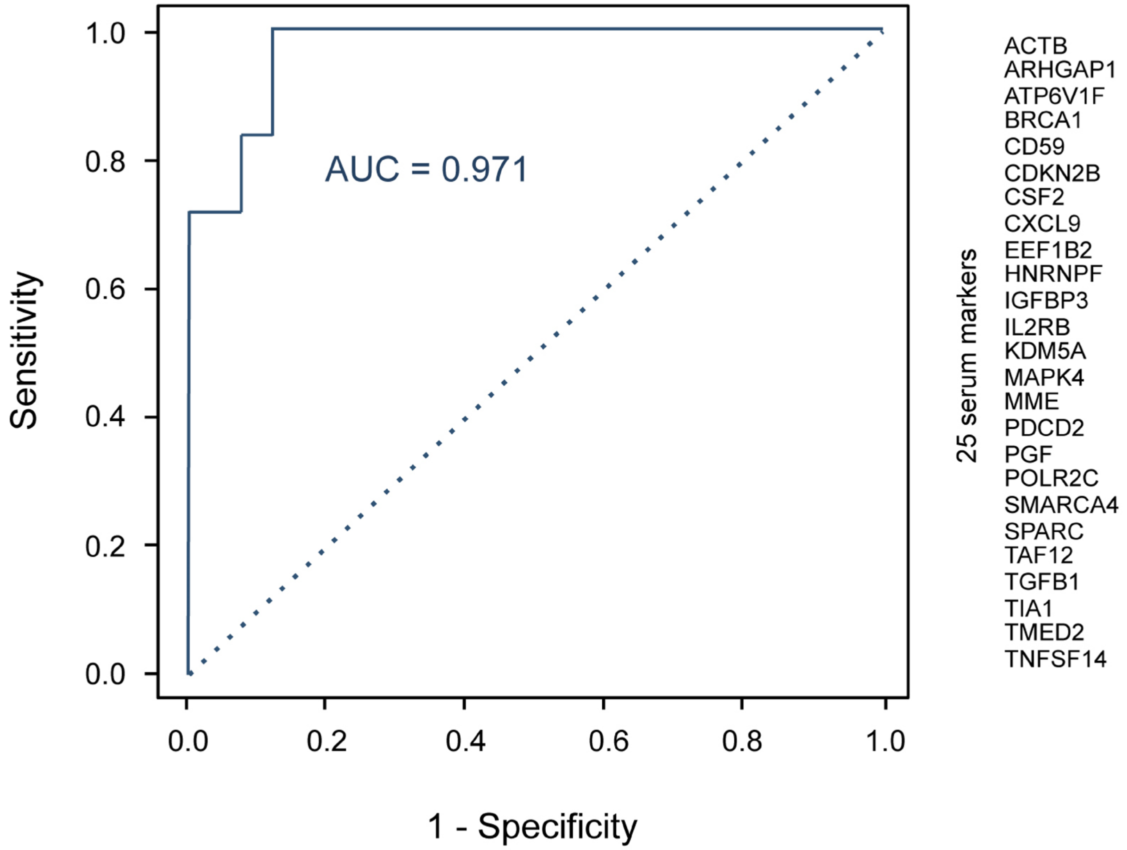

Supplementary Figure 2: Discrimination of sera from PDAV and CP patients. A ROC curve was calculated for the best performing serum proteins that distinguish PDAC from CP sera. AUC is 97.1%. The names of the 25 proteins are listed at the right margin.

Supplementary Table 1: List of targets bound by the antibodies used in the production of the small antibody microarray

See Supplementary File 1

Supplementary Table 2: The secretome profile of pancreatic cancer cell lines as compared to normal fibroblasts

See Supplementary File 2

Supplementary Table 3: Ingenuity-based functional annotation of the secretome profile of pancreatic cancer cell lines

See Supplementary File 3

Supplementary Table 4: List of antibodies used in the production of the second, larger antibody microarray. (Flags indicate multiple antibodies against the same target)

See Supplementary File 4

Supplementary Table 5: The serum proteome profile of PDAC patients (training set) as compared to healthy controls

See Supplementary File 5

Supplementary Table 6: The serum proteome profile of PDAC patients (test set) as compared to healthy controls

See Supplementary File 6
